# Rates of Bile Acid Diarrhoea After Cholecystectomy: A Multicentre Audit

**DOI:** 10.1007/s00268-021-06147-8

**Published:** 2021-05-12

**Authors:** Alexia Farrugia, Joseph Anthony Attard, Stuart Hanmer, Stuart Bullock, Siobhan McKay, Marwa Al-Azzawi, Roshneen Ali, Giles Bond-Smith, Ben Colleypriest, Sarah Dyer, Benjamin Masterman, Michael Okocha, Alan Osborne, Rikhilroy Patel, Mahmoud Sallam, Emmanuel Selveraj, Samar Shalaby, Wenrui Sun, Fraser Todd, Joel Ward, Rebecca Windle, Saboor Khan, Nigel Williams, Ramesh P. Arasaradnam

**Affiliations:** 1 Department of Gastroenterology, University Hospitals Coventry and Warwickshire NHS Trust, Coventry, CV2 2DX UK; 2 Warwick Medical School, University of Warwick, Coventry, UK; 3 Lincoln University Hospitals, Lincoln, UK; 4 Queen Elizabeth University Hospitals, Glasgow, UK; 5 Oxford University Hospitals NHS Trust, Oxford, UK; 6 Royal United Hospitals Bath NHS Trust, Avon, UK; 7 North Bristol NHS Trust, Bristol, UK; 8 School of Health and Life Sciences, Coventry University, Coventry, UK

## Abstract

**Introduction:**

Bile acid diarrhoea (BAD) can occur due to disruption to the enterohepatic circulation, e.g. following cholecystectomy. Post-cholecystectomy diarrhoea has been reported in 2.1–57.2% of patients; however, this is not necessarily due to BAD. The aim of this study was to determine the rates of bile acid diarrhoea diagnosis after cholecystectomy and to consider investigation practices.

**Methods:**

A retrospective analysis of electronic databases from five large centres detailing patients who underwent laparoscopic cholecystectomy between 2013 and 2017 was cross-referenced with a list of patients who underwent ^75^SeHCAT testing. A 7-day retention time of <15% was deemed to be positive. Patient demographics and time from surgery to investigation were collected and compared for significance (*p* < 0.05).

**Results:**

A total of 9439 patients underwent a laparoscopic cholecystectomy between 1 January 2013 and 31 December 2017 in the five centres. In total, 202 patients (2.1%) underwent investigation for diarrhoea via ^75^SeHCAT, of which 64 patients (31.6%) had a ^75^SeHCAT test result of >15%, while 62.8% of those investigated were diagnosed with bile acid diarrhoea (BAD). In total, 133 (65.8%) patients also underwent endoscopy and 74 (36.6%) patients had a CT scan. Median time from surgery to ^75^SeHCAT test was 672 days (SD ± 482 days).

**Discussion/Conclusion:**

Only a small proportion of patients, post-cholecystectomy, were investigated for diarrhoea with significant time delay to diagnosis. The true prevalence of BAD after cholecystectomy may be much higher, and clinicians need to have an increased awareness of this condition due to its amenability to treatment. ^75^SeHCAT is a useful tool for diagnosis of bile acid diarrhoea.

## Introduction

Cholecystectomy is a common surgical procedure performed for diseases of the gallbladder, commonly offered for the treatment of symptomatic gallstones [1]. However, post-operatively some patients may develop symptoms which can cause discomfort and disruption to their quality of life, one of which is diarrhoea. The frequency of diarrhoea in the post-operative period is highly variable with previous studies identifying prevalence of up to 57.2% [2–6]. The high variability within the literature is the result of most studies not being specifically powered to investigate post-cholecystectomy diarrhoea. One of the causes of post-cholecystectomy diarrhoea is bile acid diarrhoea (BAD) [7].

The British Society of Gastroenterology (BSG) guidelines for investigation of chronic diarrhoea suggest endoscopic examination and a ^75^SeHCAT scan as first-line investigations[8]. ^75^SeHCAT testing is useful to determine bile acid diarrhoea where patients who have a less than 15% retention of gamma-emitting Selenium-75-homocholic acid taurine are diagnosed with bile acid diarrhoea. This is divided into three groups, with 11–15% retention classified as mild, while 6–10% retention is moderate and less than 5% is severe. The cut-off value of 15% demonstrated a 100% sensitivity and 91% specificity [9]. While there are other ways of diagnosing BAD such as using serum C4 and faecal bile acid levels, the ^75^SeHCAT scan is more commonly used in the UK [10]. It is a condition which is amenable to treatment with bile acid sequestrants; however, it is often overlooked [10].

In this study we aimed to accurately determine the incidence of post-cholecystectomy diarrhoea across a number of hospital sites, how many patients are investigated, and how much of this is bile acid diarrhoea.

## Methods

This project was a multicentre retrospective study. Local approval was sought from the Research and Development unit of each centre separately for retrospective review of data.

An electronic retrospective database of patients undergoing laparoscopic cholecystectomy between January 2013 and December 2017 was cross-referenced with all the patients who underwent ^75^SeHCAT testing during the same time period at these centres. A 7-day^75^SeHCAT retention of less than 15% was deemed to be positive. Patient demographics were collected and compared for significance (*p* < 0.05) Mann–Whitney U test. Time from surgery to investigation was also noted, and any differences between men and women were compared using a Mann–Whitney *U* test. To further investigate this, a log of the time from cholecystectomy to investigation was taken and a Student T test was used to determine whether there were still differences in investigation times. To further quantify this difference, a regression model of time to investigation adjusted for sex was also performed. Statistical advice was sought in the data analysis.

## Results

A total of 9439 patients underwent a laparoscopic cholecystectomy between 1 January 2013 and 31 December 2017 in five UK centres: Oxford University Hospitals, North Bristol NHS Trust; Royal United Hospitals Bath NHS Trust, Queen Elizabeth University Hospital Glasgow and University Hospitals Coventry, and Warwickshire NHS Trust. Of these, 202 patients (2.1%) were investigated for BAD via ^75^SeHCAT.

### Demographic data

**Fig. 1:**
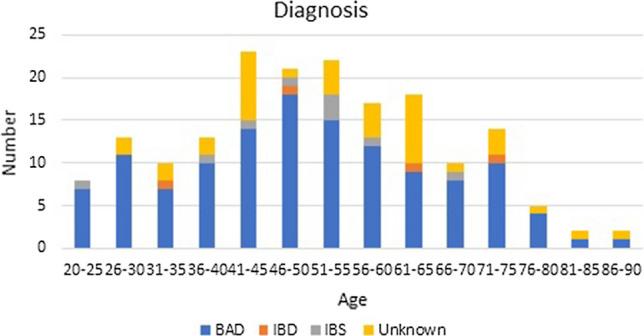
Diagnosis (*BAD* bile acid diarrhoea; *IBD* inflammatory bowel disease; *IBS* irritable bowel syndrome)

The sampled population consisted of 160 female patients (80%) and 42 male patients (20%). The age range of patients was from 20 to 90 with the highest number of patients diagnosed with BAD between the ages of 46 and 50. All patients younger than 35 were females, and the proportion of male patients increased after the age of 51. This is shown in Table 1, and the proportion of diagnosis is shown in Fig. 1.

**Table 1:** Demographics

Age	Number of patients (male:female)	Endoscopy *n* (%)	CT *n* (%)	Final diagnosis of BAD *n* (%)	Final diagnosis of IBD *n* (%)	Final diagnosis of IBS *n* (%)	Final diagnosis unknown *n* (%)
20–25	9 (0:9)	6 (2.9%)	1 (0.5%)	7 (3.5%)	0	1 (0.5%)	0
26–30	13 (0:12)	8 (3.9%)	4 (1.9%)	11 (5.4%)	0	0	2 (0.9%)
31–35	11 (0:11)	5 (2.5%)	1 (0.5%)	7 (3.5%)	1 (0.5%)	0	2 (0.9%)
36–40	17 (2:13)	9 (4.5%)	5 (2.5%)	10 (4.9%)	0	1 (0.5%)	2 (0.9%)
41–45	27 (4:23)	17 (8.4%)	8 (3.9%)	14 (6.9%)	0	1 (0.5%)	8 (3.9%)
46–50	25 (3:22)	15 (7.4%)	6 (2.9%)	18 (8.9%)	1 (0.5%)	1 (0.5%)	1 (0.5%)
51–55	26 (7:19)	16 (7.9%)	6 (2.9%)	15 (7.4%)	0	3 (1.5%)	4 (1.9%)
56–60	19 (4:15)	13 (6.4%)	6 (2.9%)	12 (5.9%)	0	1 (0.5%)	4 (1.9%)
61–65	21 (7:14)	14 (6.9%)	12 (5.9%)	9 (4.5%)	1 (0.5%)	0	8 (3.9%)
66–70	11 (4:7)	11 (5.4%)	6 (2.9%)	8 (3.9%)	0	1 (0.5%)	1 (0.5%)
71–75	15 (5:10)	11 (5.4%)	7 (3.5%)	10 (4.9%)	1 (0.5%)	0	3 (1.5%)
76–80	7 (4:3)	2 (0.9%)	5 (2.5%)	4 (1.9%)	0	0	1 (0.5%)
81–85	2 (0:2)	2 (0.9%)	2 (0.9%)	1(0.5%)	0	0	1 (0.5%)
86–90	2 (2:0)	1 (0.5%)	0	1 (0.5%)	0	0	1 (0.5%)

Of patients included in the study, 10 patients had known inflammatory bowel disease (IBD) prior to laparoscopic cholecystectomy, this being Crohn’s disease (six patients), ulcerative colitis (one patient), or indeterminate colitis (three patients). Five patients had had terminal ileal resection, only one of which had Crohn’s disease.

In total, 127 patients were diagnosed with bile acid diarrhoea (62.8% of those investigated), and four patients were newly diagnosed with IBD. Nine patients were diagnosed with IBS, and two were diagnosed as chronic pancreatic insufficiency and four as chronic cholecystitis. One patient was diagnosed with an insulinoma of the pancreas, another with Sphincter of Oddi dysfunction, one with dumping syndrome, and another with functional bowel disorder. However, 38 patients (18.8%) had a diagnosis of ‘unknown’ at the end. This is also seen in Table 1.

### Indications for ^75^SeHCAT testing

Indications for ^75^SeHCAT referral were mainly due to diarrhoea, chronic diarrhoea, loose stool, or watery stool (137 patients). In total, 21 patients were simply referred as “query of bile acid diarrhoea” or “bile acid malabsorption”. Seven patients were listed as having a change in bowel habit, and a further 17 patients reported abdominal pain, often accompanied by diarrhoea. Other reasons for referral included steatorrheoa and bloating.

### Other investigations

In total, 133 (65.8%) patients also underwent endoscopic examination (colonoscopy or flexible sigmoidoscopy) of which 86 were normal, 29 showed diverticular disease, 16 showed polyps (tubular adenomas), and two showed mild inflammation. Of those with a normal endoscopy, 43 were eventually diagnosed as having BAD.

In total, 74 (36.6%) patients had a CT scan of the abdomen and pelvis. Of these, 45 were normal, 11 showed diverticular disease, 2 demonstrated inflammatory bowel disease, and 15 showed non-bowel-related pathology.

### ^75^SeHCAT results and correlation with symptoms

The distribution of patients and their ^75^SeHCAT results is shown in Table 2. All patients had diarrhoea duration of >4 weeks. In total, 104 patients had one to five episodes per day, 34 had six to ten episodes a day, 10 patients had eleven to fifteen episodes per day, and 3 patients had more than fifteen episodes per day. For the remainder, bowel frequency was not recorded by the assessing clinician. There was no significant correlation between the ^75^SeHCAT result and the number of episodes of diarrhoea per day (*p* = 0.382, using Chi-squared test). This is also seen in Table 2.

**Table 2:** ^75^SeHCAT results and correlation with bowel habits

^75^SeHCAT results	<5%	6–10%	11–15%	>15%
Total	72	40	26	64
Male	17	11	4	10
Female	55	29	22	54
1–5 episodes/day	28	19	16	41
6–10 episodes/day	20	6	2	6
11–15 episodes/day	5	3	1	1
>15 episodes/day	2	1	0	0

### Time to investigation

**Fig. 2:**
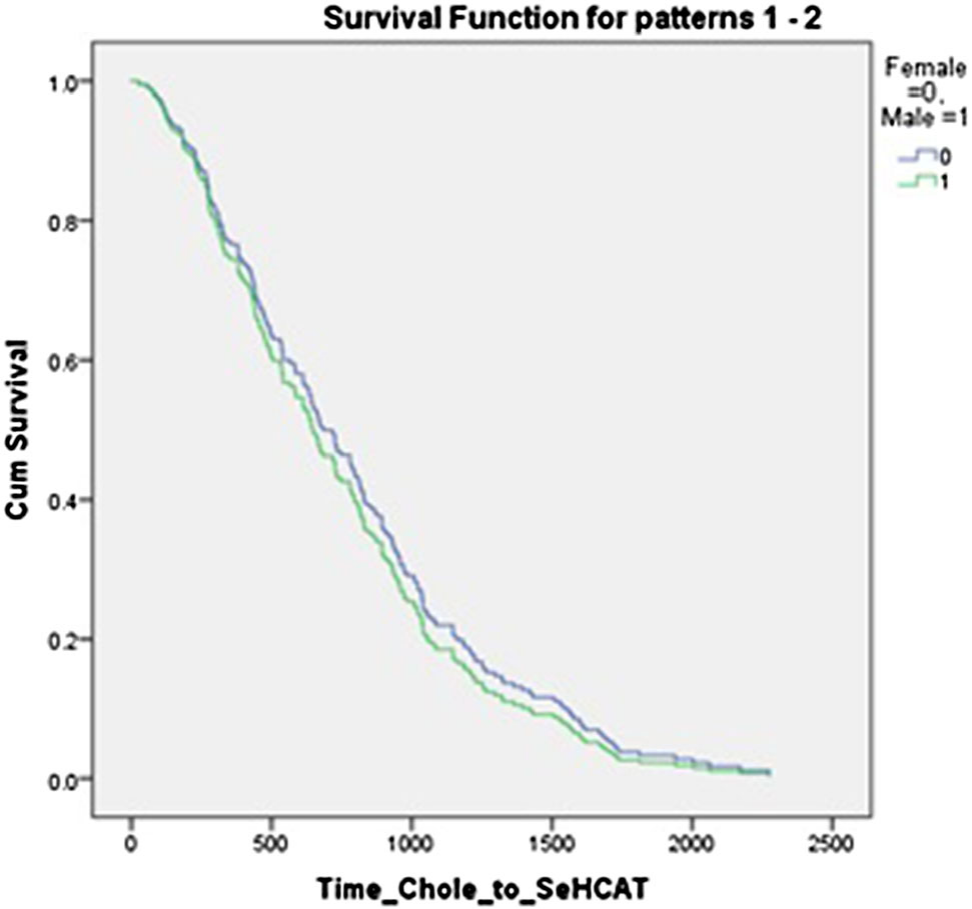
Regression analysis for time to 75 SEHCAT, adjusted for sex

**Fig. 3:**
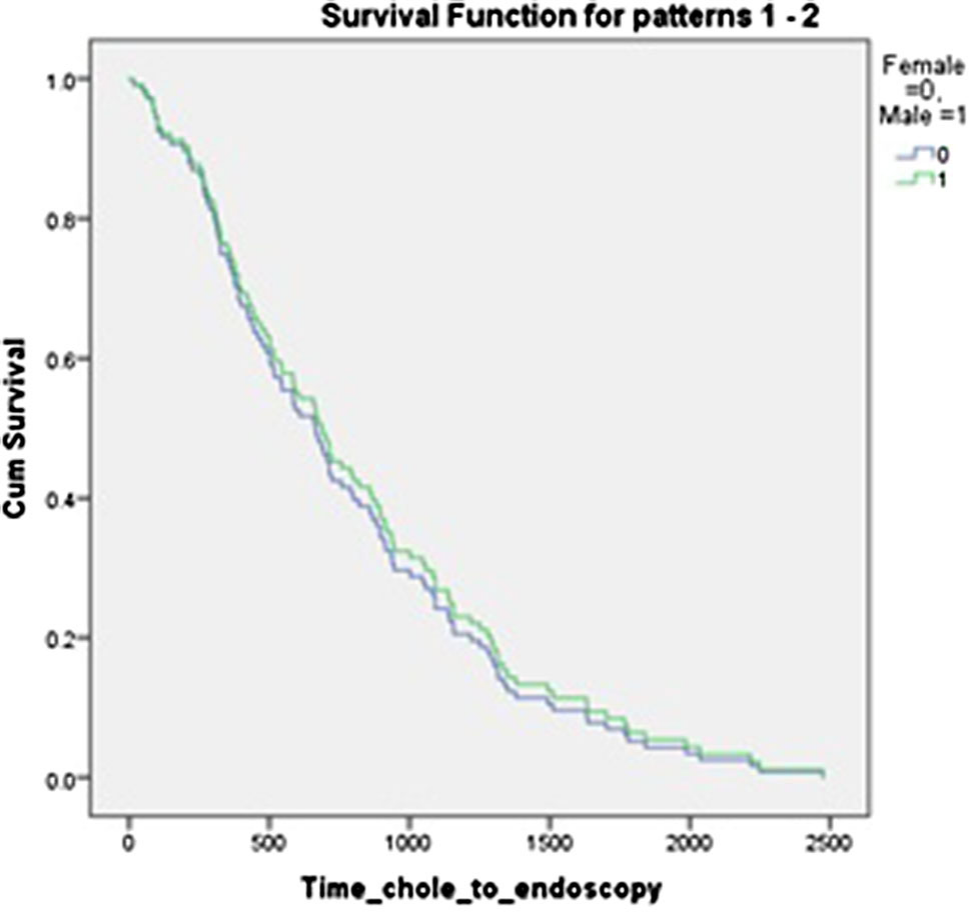
Regression analysis for time to endoscopy, adjusted for sex

**Fig. 4:**
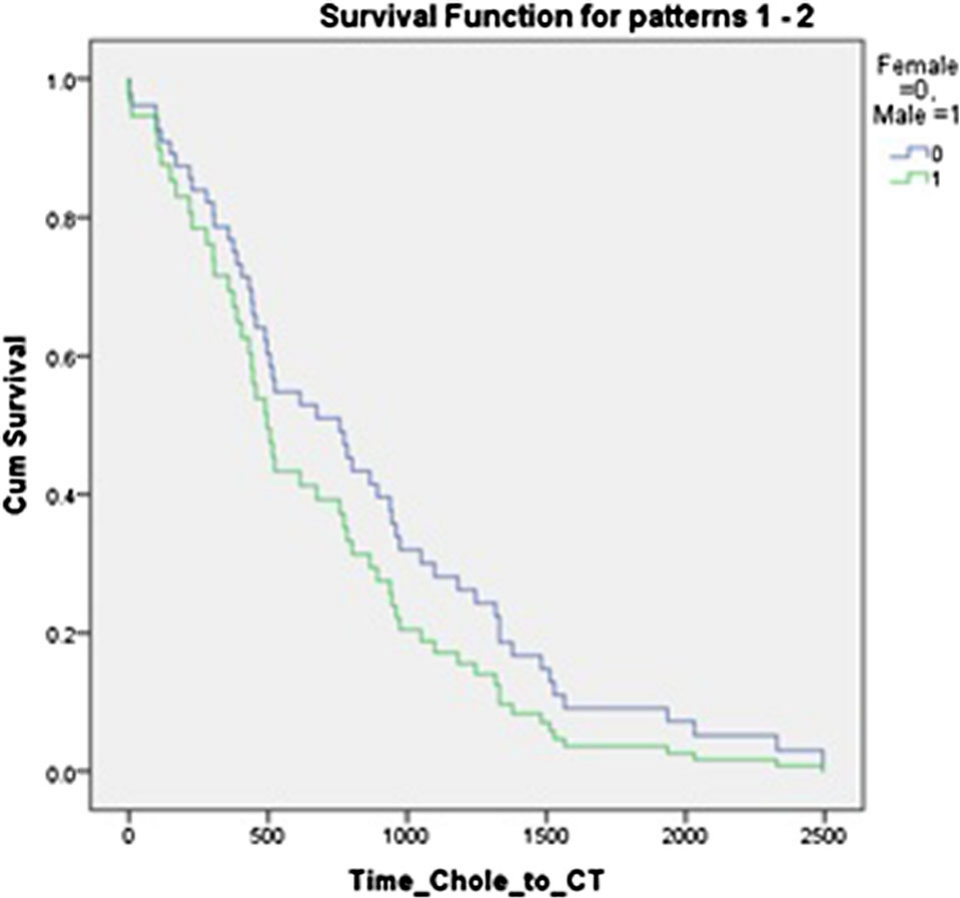
Regression analysis for time to CT, adjusted for sex

There was no significant difference between men and women in time from laparoscopic cholecystectomy to referral for ^75^ SeHCAT scan or endoscopy. There was a significant difference between referral time for men and women for CT scan (*p* = 0.022); however, this does not hold up on taking a log and performing a Students’ t test, or on performing a regression analysis adjusting for sex. This is shown in Table 3 and Figs. 2, 3, and 4

**Table 3:** Comparison of male and female median time from cholecystectomy to investigation

	Total/days (SD)	Female/days (SD)	Male/days (SD)	*p* value (Mann–Whitney *U* test)	*p* value(log and *T* test)	Regression analysis *p* value (hazard ratio with 95%CI)
^75^SeHCAT	672 (482)	726 (461)	539 (548)	0.139	0.212	0.55 (0.901; 0.63.–1.277)
Endoscopy	696 (545)	723 (517)	545 (623)	0.290	0.66	0.739 (1.078; 0.691–1.682)
CT	778 (595)	938 (531)	388 (709)	0.022	0.41	0.323 (1.39; 0.723–2.674)

## Discussion

One reason for the development of post-cholecystectomy diarrhoea is from disruption to the enterohepatic circulation, causing hepatic overproduction of bile acids. This is known as bile acid diarrhoea (BAD) of which there are three types: type one occurs secondary to ileal inflammation, thus interfering with bile acid absorption; type two is primary or idiopathic; and type three occurs secondary to other conditions where the ileum appears normal. In the latter, one of these conditions is following cholecystectomy [11, 12].

The mechanism of action to balance bile acid secretion is a negative feedback loop. Bile acid reabsorption in the ileum leads to activation of ileal FXR (farnesoid × receptor), thus inducing transcription of FGF19 (fibroblast growth factor 19) which then activates hepatic FXR. This inhibits CYP7A1 (cholesterol 7-αhydroxylase), which is the rate-limiting enzyme in bile acid synthesis, thus decreasing bile acid formation. When this is disrupted, as in BAD, there is overproduction of FGF19 leading to higher concentrations of bile acids which, in turn, leads to diarrhoea [12, 13].

In this study involving collaboration from five tertiary centres, only a small number of patients (2.1%) were investigated for diarrhoea following laparoscopic cholecystectomy. This may imply either that the rest of the patients did not require any investigation as they did not develop diarrhoea, or that their symptoms were short term and settled spontaneously without warranting medical investigation. The published literature reveals a large variation in the quoted incidence of post-cholecystectomy diarrhoea. This ranges from 2.1 to 57.2% [2–6, 14]. Our own review of the literature showed a post-cholecystectomy diarrhoea rate of 13% (Farrugia et al., Post-Cholecystectomy diarrhoea rate and predictive factors—a systematic review of the literature). Despite this, the true rate of post-cholecystectomy diarrhoea due to altered bile acid physiology has not been determined. C4 (7α-hydroxy-4-cholesten-3-one) levels, which directly correlate with bile acid synthesis, have been shown to increase following cholecystectomy, while FGF19 levels decrease [5, 15]. Despite this, the increase in C4 levels has not been shown to be related to increased frequency of bowel movements or type of stool [5].

Thus, the number of patients being investigated does not necessarily correlate with the presumed rate of post-cholecystectomy diarrhoea that is reported in the literature. This may be due to a lack of awareness that diarrhoea may develop after cholecystectomy due to faults in the pre-operative consent process. Indeed, up to 70.3% of patients are not being consented for the possibility of developing diarrhoea after laparoscopic cholecystectomy [16].

There is a clear delay in initiating investigations, with a median of 672 days between surgery and ^75^SeHCAT testing found in this study, implying that there is poor awareness within the medical community of the possibility of developing BAD after cholecystectomy. There was a difference in time to investigation between women and men, with median time to testing for female patients being 726 days while median time to testing for male patients, 539 days. While not statistically significant (*p* = 0.139), there is a median difference of 187 days. This may imply that complaints are not well regarded and in indeed one study suggests that there is a perceived reduction in constipation in women after cholecystectomy, but no real diarrhoea [17]. However, we can see from our results that it is not simply perception as patients have had positive ^75^SeHCAT tests after developing diarrhoea post-cholecystectomy.

Furthermore, we have noted that not all patients underwent endoscopic investigation in addition to ^75^SeHCAT testing, as is recommended by the British Society of Gastroenterology guidelines [8]. This could also imply that inflammatory bowel disease (IBD) was not excluded in all patients. As IBD (ileal Crohn’s) can be a cause of BAD, this is a confounding factor in our study. Another confounding factor is that some patients were known to have Crohn’s disease prior to laparoscopic cholecystectomy and others had had a previous right hemicolectomy for other conditions. As both of these factors affect the terminal ileum and may lead to bile acid malabsorption, it is unclear, for these patients, whether the BAD that developed was a consequence of malabsorption from the terminal ileum, or from bile acid overproduction following cholecystectomy, or perhaps a mixture of both. With endoscopic investigations there was an added delay of 178 days between women and men (median of 723 days for women and 545 days for men). Whilst failing to reach statistical significance (*p* = 0.29), it does represent an extra period of time with a reduced quality of life [18].

Despite CT scan being more useful in the investigation of structural rather than functional disorders, a large number of patients still had a CT scan as part of their initial investigation. In this there was a significant difference between referral time for women and men (*p* = 0.022), 938 days for women and 388 days for men. For all investigations, the median time to investigation of female patients was longer. This is a pattern that has been previously reported in other aspects of healthcare, resulting in higher morbidity and mortality for female patients [19, 20]. It is also interesting as CT scan is not recommended by the BSG guidelines for the investigation of chronic diarrhoea. However, there may have been other aspect if the clinical history led to a referral for CT scan.

Despite men being investigated (^75^SeHCAT, endoscopy and CT scan) more rapidly from initial presentation compared to women, we can still see that there is a significant delay in initiating investigations after laparoscopic cholecystectomy with a median time to investigation longer than 18 months for each investigation. Symptoms tend to develop within the first 3 months after cholecystectomy, and it is therefore apparent that these patients are not being investigated in a timely manner [21] and to the detriment of their quality of life [18]. However, there may be other issues at play such as social factors preventing some patients from seeking help or attending for tests, delays resulting from local processes such as referral practices and waiting list times for tests such as ^75^SeHCAT (which is not found in all centres) and endoscopy waiting times. As such, it is difficult to say what effect this has on time from cholecystectomy to testing. As this is a multicentre study there may also be differences in practice between regions to take into account.

This study has confirmed that the degree of BAD, as seen on the ^75^SeHCAT result, does not necessarily correlate with patient symptoms (*p* = 0.382), which is in keeping with previous work on the subject [22]. However, all patients were investigated after having diarrhoea for 4 weeks and the majority had a up to 10 episodes per day, which is congruent with the BSG guidelines for the investigation of chronic diarrhoea [8]. It is also interesting to note that whilst 62.8% of the cohort was diagnosed with BAD and 18.4% had another diagnosis, in 18.8% of patients a definitive diagnosis was not secured. This highlights that further work is required in this area to benefit this large group of patients with clinical symptoms.

We found that patients younger than 35 years of age were all females and there are generally fewer males in each age group under the age of 50. This seems to imply that younger women are at higher risk of developing PCD in our dataset. This correlates with some studies [23] but not with others that suggest younger males to be more at risk [4, 24, 25].

This study is based upon real-time linked clinical data, thus showing the true perspective of patients who were investigated post-laparoscopic cholecystectomy for diarrhoea. Patients who were empirically started on bile acid sequestrants rather than being investigated via ^75^SeHCAT would not have been captured in the present study. Another possible limitation is that not all patients who develop diarrhoea are investigated via ^75^SeHCAT; thus, the true numerator remains unknown. BAD is not a well-known condition, and therefore, the only patients who were referred for ^75^SeHCAT testing were those seen by GPs, physicians, and surgeons who are aware of the condition. We also have no data regarding response to treatment in these patients identified here who were diagnosed with BAD. We have identified a large discrepancy between the number of male and female patients within our dataset, as such there may be an element of selection bias. However, the advantage of this study is that it is a multicentre study using ^75^SeCHAT as the investigation of choice with defined cut-off values for diagnosis of BAD. It also benchmarks the current clinical scenario when it comes to the investigation of chronic diarrhoea after cholecystectomy. While this is the largest study of its kind to date, further studies involving direct comparison between those patients investigated, and those who are not, for diarrhoea following cholecystectomy would present a more comprehensive picture of this difficult condition and would not only improve our understanding but allow for improved patient care.

## Conclusion

A small proportion of post-cholecystectomy patients were investigated for BAD (2.1%), and in those that were investigated 62.8% were positive for BAD as indicated by ^75^SeHCAT testing (^75^SeHCAT results <15%). There was a significant time delay to diagnosis following the onset of symptoms. This may in part be because cholecystectomy is mostly undertaken as a day case procedure and routine follow-up is rarely required. The true prevalence of BAD post-cholecystectomy may be much higher, and clinicians in both primary and secondary care need to have an increased awareness of this condition due to its amenability to treatment. Other options including serum C4 and faecal measurements of bile acid remain alternatives where^75^SeHCAT is unavailable.
